# Pathogen Virulence Impedes Mutualist*-*Mediated Enhancement of Host Juvenile Growth via Inhibition of Protein Digestion

**DOI:** 10.1016/j.chom.2015.09.001

**Published:** 2015-10-14

**Authors:** Berra Erkosar, Gilles Storelli, Mélanie Mitchell, Loan Bozonnet, Noémie Bozonnet, François Leulier

**Affiliations:** 1 Institut de Génomique Fonctionnelle de Lyon, Ecole Normale Supérieure de Lyon, Centre National de la Recherche Scientifique, Université Claude Bernard Lyon 1, Unité Mixte de Recherche 5242, 69364 Lyon, Cedex 07, France

## Abstract

The microbial environment impacts many aspects of metazoan physiology through largely undefined molecular mechanisms. The commensal strain *Lactobacillus plantarum*^*WJL*^ (*Lp*^*WJL*^) sustains *Drosophila* hormonal signals that coordinate systemic growth and maturation of the fly. Here we examine the underlying mechanisms driving these processes and show that *Lp*^*WJL*^ promotes intestinal peptidase expression, leading to increased intestinal proteolytic activity, enhanced dietary protein digestion, and increased host amino acid levels. *Lp*^*WJL*^-mediated peptidase upregulation is partly driven by the peptidoglycan recognition and signaling cascade PGRP-LE/Imd/Relish. Additionally, this mutualist-mediated physiological benefit is antagonized upon pathogen infection. Pathogen virulence selectively impedes *Lp*^*WJL*^-mediated intestinal peptidase activity enhancement and juvenile growth promotion but does not alter growth of germ-free animals. Our study reveals the adaptability of host physiology to the microbial environment, whereby upon acute infection the host switches to pathogen-mediated host immune defense at the expense of mutualist-mediated growth promotion.

## Highlights

• *Lactobacillus plantarum*^*WJL*^ sustains gut peptidase expression in *Drosophila*
• *Lp*^*WJL*^ association enhances dietary protein digestion and host amino acid levels
• Foodborne infection impedes *Lp*^*WJL*^-mediated intestinal peptidase activity
• Pathogen virulence impedes *Lp*^*WJL*^-mediated promotion of juvenile growth

## Graphical Abstract

**Figure:**
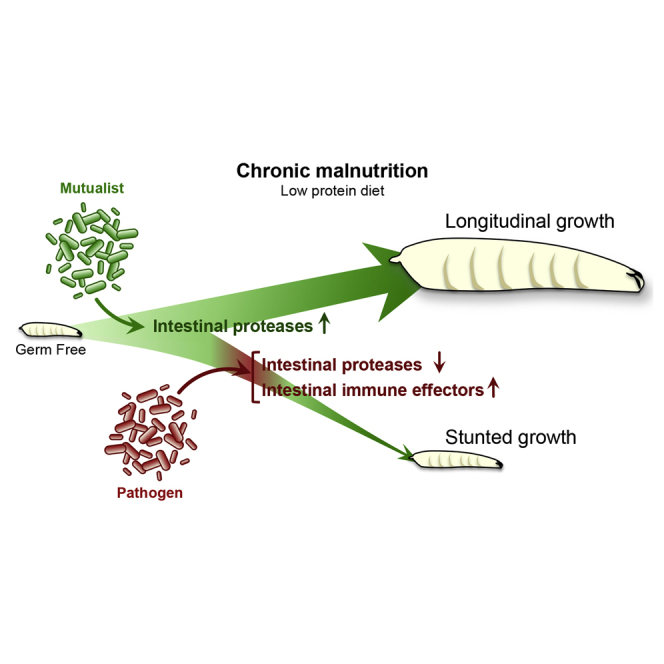

## Teaser

The microbial environment impacts animal physiology. Erkosar et al. reveal that commensal *Lactobacillus plantarum*^*WJL*^ enhances *Drosophila* growth by promoting intestinal peptidase expression, thereby increasing protein digestion and amino acid levels. However, acute infection impedes this physiological benefit, suggesting that the host activates immune defense at the expense of mutualist-mediated growth promotion.

## Introduction

Metazoans establish diverse forms of functional interactions with their microbial environment, and such interactions contribute to many aspects of animal development, physiology, and evolution (McFall-Ngai et al., 2013). Mutualistic interactions are a type of symbiosis where both partners bring reciprocal functional benefits to each other. In contrast, in pathogenic or parasitic interactions, the association is beneficial for one partner and deleterious for the other (Douglas, 2010). Despite recent progress, the molecular mechanisms through which the microbial environment exerts its beneficial influences on animal biology are still largely undefined.

*Drosophila melanogaster* has recently emerged as a powerful model organism to study beneficial host-bacteria interactions (Erkosar et al., 2013; Lee and Brey, 2013). *Drosophila* is associated with bacterial communities of low complexity composed of a handful of dominant species (mostly of the *Acetobacteraceae* and *Lactobacillaceae* families). The powerful genetic tools in *Drosophila*, coupled to the simplicity to cultivate germ-free (GF) animals and to manipulate its commensal bacterial species makes *Drosophila* an ideal host model to study the molecular mechanisms underlying bacteria-mediated physiological benefits. Commensal bacteria affect *Drosophila* biology throughout its life cycle (Buchon et al., 2013; Erkosar et al., 2013; Lee and Brey, 2013). In adults, they influence lifespan (Brummel et al., 2004; Guo et al., 2014; Ren et al., 2007), dictate host nutrition and metabolic responses (Wong et al., 2014; Yamada et al., 2015), shape mating preference (Sharon et al., 2010), mediate social attraction (Venu et al., 2014), increase host resistance to several intestinal pathogens (Blum et al., 2013), modulate intestinal immune homeostasis (Bosco-Drayon et al., 2012; Lhocine et al., 2008; Paredes et al., 2011), and promote intestinal epithelium renewal (Buchon et al., 2009a; Shin et al., 2011). During the juvenile phase (i.e., from the end of embryogenesis until metamorphosis), commensal microbes sustain larval growth and maturation rate, a feature even more pronounced when the host is facing undernutrition (Shin et al., 2011; Storelli et al., 2011).

We previously showed that a selected strain of *Lactobacillus plantarum* isolated from *Drosophila* intestine, *Lactobacillus plantarum*^*WJL*^ (*Lp*^*WJL*^) (Ryu et al., 2008), is sufficient on its own to recapitulate the beneficial effect of more complex *Drosophila*-associated bacterial communities on host juvenile growth, especially upon undernutrition (Storelli et al., 2011). Using this monoxenic animal model (one microbe-one host), we revealed that *Lp*^*WJL*^ exerts its beneficial activity by acting upstream of hormonal signals known to coordinate *Drosophila* systemic growth and maturation rates. Indeed, we demonstrated that *Lp*^*WJL*^ association requires optimal mTOR signaling activity in the fat-body to enhance the activity of *Drosophila* insulin-like peptides (dILPs) and in the prothoracic gland for ecdysone production to promote growth and maturation. In addition, we showed that *slimfast* expression is necessary in the fat-body for growth promotion upon *Lp*^*WJL*^ association (Storelli et al., 2011). *Slimfast* encodes a transporter for the intracellular uptake of circulating amino acids (AAs), which are the main activators of TOR kinase activity. We therefore proposed that *Lp*^*WJL*^ may exert its growth-promoting effect by enhancing protein assimilation in the host to promote optimal AAs availability that triggers mTOR signaling activity and subsequently sustains systemic growth and maturation (Storelli et al., 2011).

In this manuscript, we directly address this hypothesis and show that *Lp*^*WJL*^ association sustains intestinal peptidase expression, partly via the PGRP-LE/Imd/Relish signaling cascade, which translates into increased intestinal proteolytic activity, enhanced protein digestion, and improved AA levels in the host. In addition, we reveal that this mutualist-mediated physiological benefit is antagonized upon foodborne pathogen infection.

## Results

### *Lactobacillus plantarum* Association Sustains Intestinal Peptidase Gene Expression during Juvenile Growth

**Figure 1:**
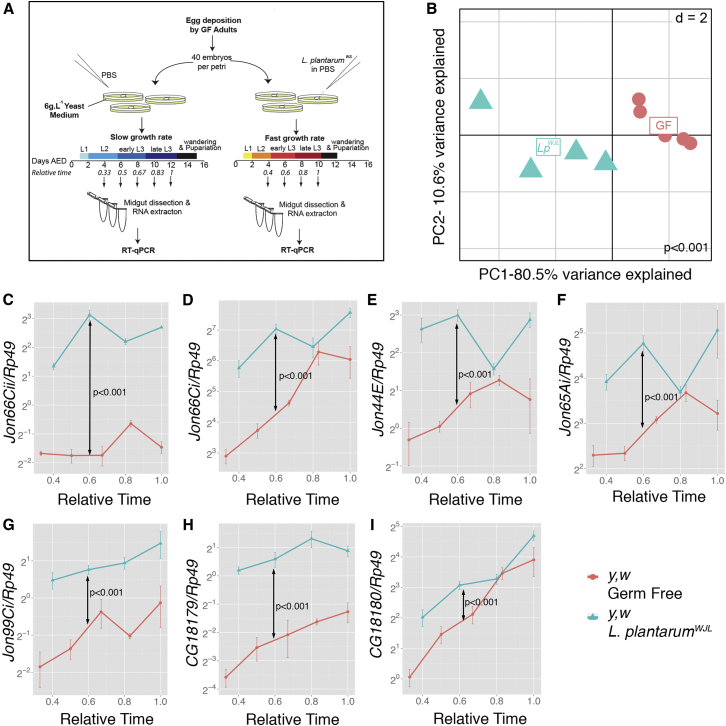
*L. plantarum* Association Sustains Intestinal Peptidase Gene Expression during Juvenile Growth (A) Experimental setup for the RT-qPCR analysis. Actual and relative developmental timings and developmental stages are indicated for GF and *Lp*^*WJL*^-associated animals; the emergence of the first white pupae is used as an anchor for relative timings in each condition (1 = day 10 AED for *Lp*^*WJL*^ condition and D12 for GF condition). AED: after egg deposition. (B) Projection of the RT-qPCR dataset into the space of the first and second PCs. d: size of the background grid. (C–I) Mean ± SEM of ΔCt^*gene*^*/*ΔCt^*rp49*^ ratios for (C) *Jon66Cii*, (D) *Jon66Ci*, (E) *Jon44E*, (F) *Jon65Ai*, (G) *Jon99Ci*, (H) *CG18179*, and (I) *CG18180* detected in midguts of GF (red) and *Lp*^*WJL*^*-*associated (blue) larvae along larval development. p values of the MANOVA analysis from all variables (B) and gene-specific two-way ANOVA ([C] –[I]) are given (“Bacteria” effect only). See also Tables S1 and S2.

Previously, we studied changes in host transcriptome in adult flies associated with a cocktail of commensal bacterial strains and detected several peptidase genes whose expressions are significantly upregulated as compared their GF siblings (Erkosar et al., 2014). Using the FlyAtlas tool, we identified that most of these peptidases have a basal expression level enriched in both the adult and larval midguts of the conventional individuals (CONV; i.e, carrying commensal microbes), a signature confirmed in adult midguts by RT-qPCR (Erkosar et al., 2014). Since we hypothesized that *Lp*^*WJL*^ may exert its growth-promoting activity during juvenile development by enhancing protein assimilation in the host (Storelli et al., 2011), this observation prompted us to investigate the expression of these peptidase genes by time course RT-qPCR in larval midguts of GF or *Lp*^*WJL*^-associated individuals on a low-nutrition diet (see Figure 1A for detailed experimental scheme). We found the expression profile of 7 peptidases (*Jon66Cii*, *Jon66Ci*, *Jon44E*, *Jon65Ai*, *Jon99Ci*, *CG18179*, and *CG18180*) presents a robust and detectable transcriptional signature during larval development (Figures 1B–1I). Principal-component analysis (PCA) on the whole dataset (expression levels of the seven genes at all time points in both conditions) reveals that PC1, which explains 80.5% of the observed variance, is enough to separate the GF and *Lp*^*WJL*^ groups. Such separation indicates that peptidase expression levels in the midguts of *Lp*^*WJL*^-associated larvae remarkably differ from the GF condition (Figure 1B). This difference is also strongly significant when the whole dataset is analyzed by multivariate analysis of variance (MANOVA-Bacteria: p < 0,001; Figure 1B; Table S1); furthermore, we compared each of the seven peptidases in GF and *Lp*^*WJL*^ larvae during the same defined period of growth and found that the expression of any given peptidase is significantly elevated in *Lp*^*WJL*^ animals along most of the time points (Figures 1C–1I and ANOVA-Bacteria; Table S1). Notably, the peptidase genes expression tends to increase during larval development even in GF condition (MANOVA-Time and ANOVA-Time and GF Group:Time; Table S1), but the trend of increase is only marginally affected by the bacterial association (MANOVA-Bacteria × Time and ANOVA-Bacteria × Time; Table S1). Taken together, these results demonstrate that *Lp*^*WJL*^ association triggers an overall increase in the expression levels of several intestinal peptidases but does not alter their expression dynamics during juvenile growth.

### *Lactobacillus plantarum* Association Enhances Intestinal Proteolytic Activity, which Is Necessary and Sufficient for Juvenile Growth Promotion

**Figure 2:**
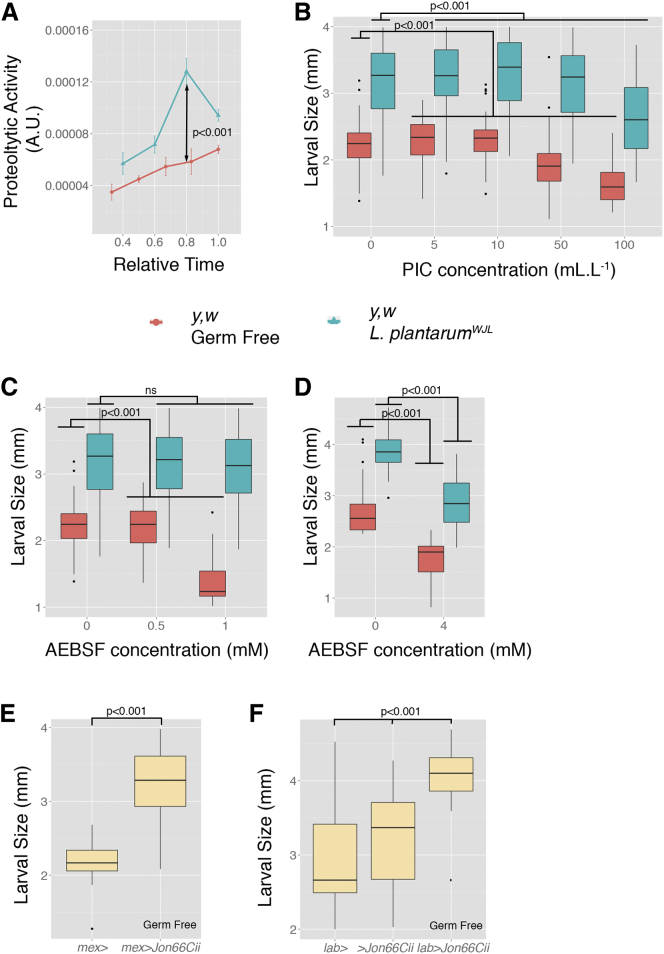
*L. plantarum* Association Enhances Intestinal Proteolytic Activity that Is Necessary for Juvenile Growth (A) Proteolytic activity (mean ± SEM) detected in dissected midguts from GF or *Lp*^*WJL*^-associated animals during larval development. Proteolytic activity is measured by azocasein assay normalized to total protein quantity for each sample; a.u. are used. (B–D) Longitudinal size of larvae (Boxplots, n > 23) measured 6 days AED on the low-nutrition diet containing increasing quantities of complete PIC (B) or the serine protease inhibitor 4-(2-AminoEthyl) benzenesulfonyl fluoride hydrochloride (AEBSF) ([C] and [D]). (E and F) Longitudinal size of larvae (Boxplots, n > 19) measured 7 days AED on the low-nutrition diet. Genotypes used: (E) *mex*>: *mex*-GAL4 - *mex*>*Jon66Cii*: *mex*-GAL4/+;;UAS-*Jon66Cii*-3xHA/+. (F) *lab*>: *lab*-GAL4 - *lab*>*Jon66Cii*: *lab*-GAL4/+;;UAS-*Jon66Cii*-3xHA/+ − > *Jon66Cii*: UAS-*Jon66Cii*-3xHA. p values obtained from two-way (only “Bacteria” effect is shown [A] and one-way ANOVA, [B]–[D] and [F]), and Student’s t est with Welch correction (E) are indicated. Results of Tukey’s post hoc pairwise comparisons confirmed the statistical significance observed in (F). See also Figure S1 and Table S1.

Next, we measured intestinal proteolytic activity upon *Lp*^*WJL*^ association. To this end, we dissected midguts from either GF or *Lp*^*WJL*^-associated larvae grown on low-nutrition diet, and assayed intestinal proteolytic activity over time. Similarly to the expression profile of the peptidase genes delineated in Figure 1, the intestinal protease activities in GF larval midguts increase steadily during the period tested, and upon *Lp*^*WJL*^ association, the intestinal proteolytic activities are consistently higher than that in the GF animals (Figure 2A). To rule out the possibility that such observed increase of proteolytic activities are the result of a mere addition of the bacterial protease activity to the system, we monitored the proteolytic activities of live *Lp*^*WJL*^ cells and in the supernatant harvest from an over-night culture. With 10^9^ *Lp*^*WJL*^ cells, a quantity several logs above the bacterial load detected in *Lp*^*WJL*^-associated midguts (see Figure 6D) or in the supernatant, we detected marginal proteolytic activities using the same biochemical assay as compared to the activity detected from either GF or *Lp*^*WJL*^-associated midguts (Figure S1).

We then questioned the functional importance of such intestinal proteolytic activity for *Drosophila* juvenile growth. To this end, we grew *Drosophila* GF larvae on the low-nutrition diet mixed with increasing quantities of either a complete Protease Inhibitor Cocktail (PIC) or a specific irreversible serine-protease inhibitor (AEBSF) and quantified the length of all individual larvae fed on such diet 7 days after egg deposition (AED). The growth rate of GF larvae was reduced, resulting in smaller larvae at day 7 AED as the quantities of protease inhibitors in their diet increase (Figures 2B–2D). Interestingly, *Lp*^*WJL*^ association, which enhances peptidase expression and activity, buffers the deleterious effect of protease inhibitors on juvenile growth, as exemplified by a reduced sensitivity to protease inhibitors of *Lp*^*WJL*^-associated larvae (Figures 2B and 2C), yet at higher protease inhibitors concentration, *Lp*^*WJL*^-mediated growth promotion was also diminished (Figures 2B and 2D). Our results therefore demonstrate that *Lp*^*WJL*^ association sustains intestinal protease activity and that intestinal peptidase activity is required for juvenile growth in general and *Lp*^*WJL*^-mediated growth promotion in particular.

Next, we tested if the induced expression of an intestinal protease is sufficient to trigger larval growth. To this end, we directed the ectopic expression of *Jon66Cii* (the protease most differentially expressed upon *Lp*^*WJL*^ association) in the midgut of young GF larvae and assayed their longitudinal growth. We observed a marked growth promotion upon *Jon66Cii* induction in the enterocytes of both the entire midgut (*mex*-GAL4; Figure 2E) (Phillips and Thomas, 2006) and of the acidic region of the middle midgut (*labial*-GAL4; Figure 2F) (Hoppler and Bienz, 1994). These results therefore demonstrate that intestinal peptidase expression is sufficient to trigger juvenile growth. Collectively, our results demonstrate that *Lp*^*WJL*^ association enhances intestinal protease expression and activity, which is necessary and sufficient for juvenile growth promotion upon undernutrition.

### *Lactobacillus plantarum* Association Promotes Protein Digestion and Sustains AA Levels during Juvenile Growth

**Figure 3:**
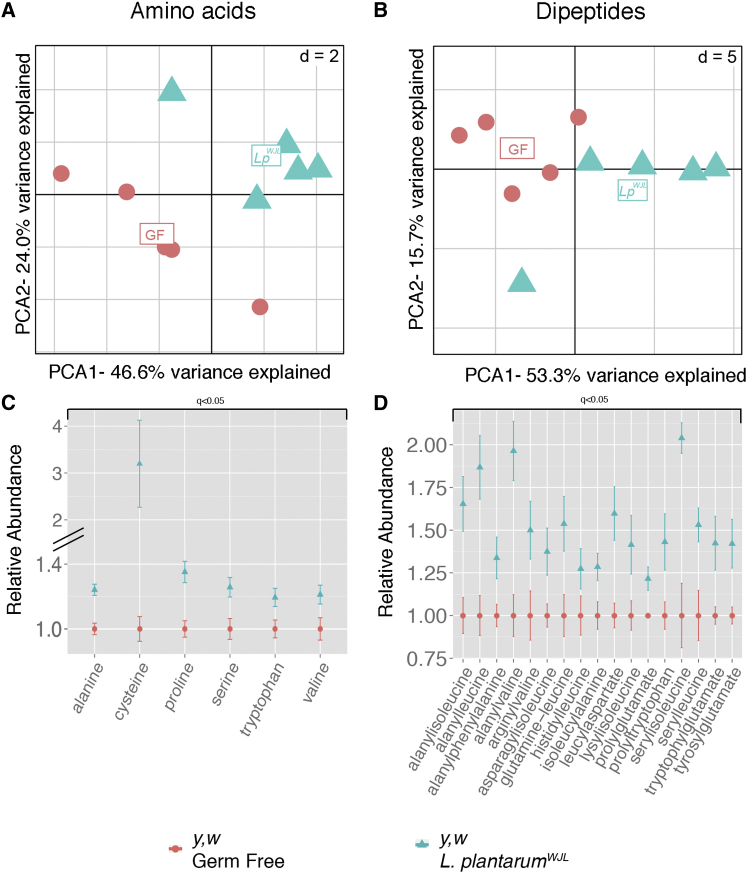
*L. plantarum* Association Promotes Protein Digestion and AAs Levels in the Larvae (A and B) Projection of free AAs (A) and dipeptides levels (B) into the space of the first and second PCs. Free AA levels in GF and *Lp*^*WJL*^-associated animals cluster separately from each other, although one PC is not sufficient to explain the variance due to inter-group variability (A). Variance in dipeptide levels is mainly explained by PC1 (B). The separation between the two groups is clear, but the distance within groups is not elevated, highlighting the small amplitude of the levels difference observed in (D). d: size of the background grid. (C and D) Relative abundance (mean ± SEM) of AAs (C) and dipeptides (D) that are significantly higher in *Lp*^*WJL*^*-*associated larvae compared to GF larvae. p values are obtained from comparison of means from GF and *Lp*^*WJL*^*-*associated larvae by Student’s t Test with Welch correction, and q values are calculated upon FDR correction. See also Table S1.

We next questioned the physiological consequences of *Lp*^*WJL*^-mediated enhanced intestinal proteolytic activity and wondered if that phenomenon increases protein digestion and free AAs levels in the host. To this end, we analyzed the relevant data from a global metabolomic study of GF and *Lp*^*WJL*^ larvae raised for 2.5 days AED on the low-nutrition diet (G.S. and F.L., unpublished data). We focused on dipeptides and free AAs since these metabolites are the end-results of protein digestion process. 21 AAs and 50 dipeptides were detected and quantified in the lysates of GF and *Lp*^*WJL*^ larvae (Table S1). A PCA analysis of this dataset tends to group the biological replicates generated from the same condition and separates the samples according to the bacterial association for either the free AAs (Figure 3A) or the dipeptides datasets (Figure 3B). Interestingly, the levels of most free AAs, including essential AAs, are increased in *Lp*^*WJL*^ larvae (Table S1). Particularly, alanine, cysteine, proline, serine, tryptophan, and valine are significantly increased (Figure 3C). Similarly, most dipeptides accumulate in *Lp*^*WJL*^ larvae, and 17 of them reach statistical significance (Table S1; Figure 3D). These observations strongly suggest that *Lp*^*WJL*^ association increases protein digestion to increase host’s AAs levels.

### PGRP-LE/Imd/Relish Pathway Partly Regulates *Lactobacillus plantarum*-Mediated Intestinal Peptidase Gene Expression during Juvenile Growth

**Figure 4:**
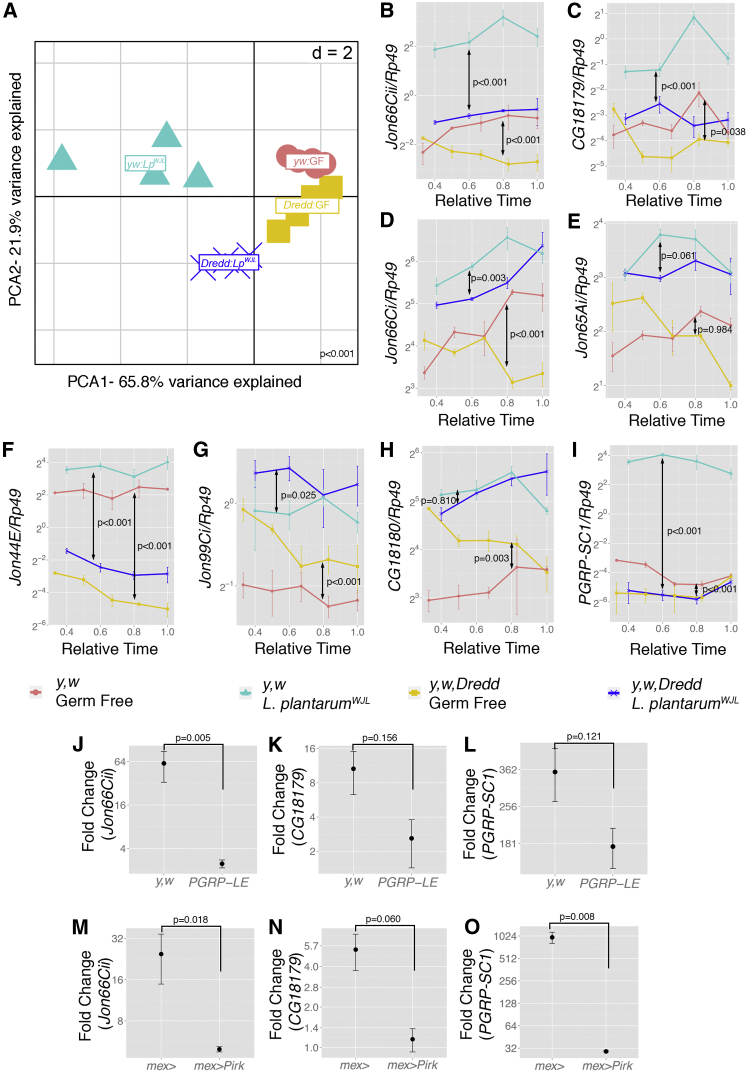
PGRP-LE/Imd/Relish Pathway Partly Regulates *L.plantarum*-Mediated Intestinal Peptidase Gene Expression during Juvenile Growth (A) Projection of RT-qPCR dataset into the space of the first and second PCs. Note that expression levels in *Lp*^*WJL*^*-*associated wild-type larvae clusters separately from the other conditions. PC1 separates *Lp*^*WJL*^ versus GF groups, whereas PC2 separates the two genotypes. yw: *y*,*w* genotype; Dredd: *y*,*w*,*Dredd* genotype; GF: germ free; *Lp*^*WJL*^: *L. plantarum*^*WJL*^ associated. (B–I) Mean ± SEM of ΔCt^*gene*^*/*ΔCt^*rp49*^ ratios for (B) *Jon66Cii*, (C) *CG18179*, (D) *Jon66Ci*, (E) *Jon65Ai*, (F) *Jon44E*, (G) *Jon99Ci*, (H) *CG18180*, and (I) *PGRP-SC1a/b* detected in midguts of wild-type GF (red) and *Lp*^*WJL*^*-*associated (light blue) larvae or *Dredd* mutant GF (yellow) and *Lp*^*WJL*^*-*associated (dark blue) larvae along larval development. p values obtained from MANOVA (“Bacteria” and “Genotype” interaction) analyzing all variables (A) and from gene-specific two-way ANOVA (“Genotype” effect in *Lp*^*WJL*^ and GF groups; [B] –[I]) are indicated. (J–L) Fold changes of mean ± SEM of ΔCt^*gene*^*/*ΔCt^*rp49*^ ratios for (J) *Jon66Cii*, (K) *CG18179*, and (L) *PGRP-SC1a/b* detected in midguts of wild-type or *PGRP-LE* mutant larvae associated with *Lp*^*WJL*^ (day 8 AED) relative to the size-matched germ free individuals (day 12 AED). (M–O) Fold changes of mean ± SEM of ΔCt^*gene*^*/*ΔCt^*rp49*^ ratios for (M) *Jon66Cii*, (N) *CG18179*, and (O) *PGRP-SC1a/b* detected in midguts of wild-type (*mex*>: *mex*-GAL4/+) or *mex*>*Pirk* (*mex*-GAL4/UAS-*Pirk)* larvae associated with *Lp*^*WJL*^ (day 8 AED) relative to the size-matched GF individuals (day 12 AED). p values obtained from two-way ANOVA are indicated (“Bacteria” and “Genotype” interaction; [J]–[O]). See also Tables S1 and S2.

We next wondered how *Lp*^*WJL*^ association triggers the upregulation of the host intestinal peptidases. To tackle this question, we analyzed the expression dynamics of the peptidases genes influenced by *Lp*^*WJL*^ association in a mutant background where the host transcriptomic response to the microbial environment is impaired, namely in *Dredd* mutants. Loss of Dredd results in complete loss of function of the Imd/Relish signaling pathway (Leulier et al., 2000). This signaling cascade, which is triggered by the direct sensing of peptidoglycan fragments of bacterial origin, is the prerequisite to induce the expression of hundreds of immune-regulated genes (De Gregorio et al., 2002), mount efficient immune responses to infectious bacteria (Myllymäki et al., 2014), and trigger immune tolerance to commensal microbes (Bosco-Drayon et al., 2012; Lhocine et al., 2008; Paredes et al., 2011). Importantly, we and others have recently demonstrated the central role of the Imd/Relish cascade activity to promote the expression of many microbiota-regulated genes in the adult midgut (Broderick et al., 2014; Erkosar et al., 2014). We therefore analyzed the expression levels of the peptidases in the midguts of wild-type and *Dredd* mutant larvae during juvenile growth on the low-nutrition diet in either GF or *Lp*^*WJL*^-associated conditions (Figures 4A–4H). As a control, we quantified the expression of *PGRP-SC1a/b*, a known microbiota-regulated gene whose induction in the larval midgut upon *Lp*^*WJL*^ association is dependent on the Imd/Relish cascade activity (Figure 4I) (Bosco-Drayon et al., 2012). We first analyzed the entire expression dataset (for all time points, genotypes, and conditions) by projecting the expression results on two principal components (PCs) (Figure 4A). This PCA clearly singled out the *Lp*^*WJL*^ association effect on peptidase expression in wild-type animals (as seen in Figure 1C). Of note, *Lp*^*WJL*^-associated peptidase expression data points grouped in two distant pools separated by genotype (WT versus *Dredd*), while the GF individuals data grouped closer. This signature indicates that Dredd governs peptidase expression in the larval midgut, with a marked effect upon *Lp*^*WJL*^ association. The statistical analysis of the whole dataset confirms this observation and reveals a very strong statistical interaction between bacterial association and genotype (Figure 4A and MANOVA-Bacteria × Genotype; Table S1). In addition, we further analyzed the time course expression profiles of individual peptidase genes with or without *Lp*^*WJL*^ in different genetic background (Figures 4B–4H). First, the expression levels of *Jon66Cii*, *CG18179*, and *PGRP-SC1a/b* in both GF and *Lp*^*WJL*^-associated animals are *Dredd* dependent, and loss of Dredd markedly dampens the *Lp*^*WJL*^-mediated enhancement of expression of these peptidases to a level similar to that of the wild-type GF animals (Figures 4B, 4C, and 4I). The basal expression level (i.e., in GF) and *Lp*^*WJL*^-mediated induction of *Jon66Ci* and *Jon65Ai* only exhibit a partial *Dredd* dependency (Figures 4D and 4E). In contrast, *Jon44E*, *Jon99Ci*, and *CG18180* induction upon *Lp*^*WJL*^ association is moderately *Dredd* dependent, despite statistical significance being reached (*Jon44E* and *Jon99Ci*; Figures 4F and 4G*)* or even *Dredd* independent (Figure 4H). Finally, the basal expression level of *Jon44E* is reduced in *Dredd* mutant, while basal expression levels of *Jon99Ci* and *CG18180* are increased in the same context (Figures 4G and 4H). Taken together, these gene specific signatures illustrate the complexity of the intestinal peptidase gene regulation. Yet our results clearly demonstrate that the activity of the Imd/Relish cascade is necessary to mediate the expression of several peptidases in the larval midgut upon *Lp*^*WJL*^ association.

We next focused on *Jon66Cii* and *CG18179*, whose induction is strong upon *Lp*^*WJL*^ association and markedly *Dredd* dependent, and tested if PGRP-LE, one of the pattern recognition receptor acting upstream of the Imd/Relish signaling cascade (Kaneko et al., 2006; Takehana et al., 2004) is necessary for the induction of these two peptidases upon *Lp*^*WJL*^ association. Similarly to *PGRP-SC1a/b* (Bosco-Drayon et al., 2012) (Figure 4L), *Jon66Cii* and *CG18179* induction upon *Lp*^*WJL*^ association was strongly impaired in *PGRP-LE* mutant larvae (Figures 4J and 4K). PGRP-LE is enriched in both adult and larval midgut (Neyen et al., 2012) and has been proposed to function as a direct peptidoglycan sensor in the adult and larval enterocytes (Bosco-Drayon et al., 2012; Neyen et al., 2012). We therefore wanted to test if *PGRP-SC1a/b*, *Jon66Cii*, and *CG18179* induction upon *Lp*^*WJL*^ association requires the PGRP-LE/Imd/Relish cascade activity specifically in the enterocytes. Pirk is an inhibitor of this signaling cascade that alters Imd activation by PGRPs (Aggarwal et al., 2008; Kleino et al., 2008; Lhocine et al., 2008). We thus selectively overexpressed Pirk in enterocytes with *mex*-GAL4 and observed that *Lp*^*WJL*^-mediated induction of *Jon66Cii*, *CG18179*, and *PGRP-SC1a/b* is markedly reduced (Figures 4M–4O). Therefore, the PGRP-LE/Imd/Relish pathway activity is required in enterocytes to mediate the induction of selected intestinal peptidase gene upon *Lp*^*WJL*^ association.

Having demonstrated that the *Lp*^*WJL*^-mediated peptidase expression enhancement is at least partly Dredd dependent and requires the PGRP-LE/Imd/Relish pathway activity in the enterocytes, we detected no alteration of the intestinal proteolytic activity of *Dredd* mutant larval midguts in either GF or *Lp*^*WJL*^-associated condition (Figures S2A–S2C) or any marked impact of *Dredd* loss of function on *Lp*^*WJL*^-mediated juvenile growth promotion (Figure S2D). These results indicate that the *Dredd* dependency is partial and not rate limiting for *Lp*^*WJL*^-mediated intestinal peptidase activity and juvenile growth promotion and suggest a more complex regulation of peptidase expression.

### Infection by Foodborne Pathogen Antagonizes *Lactobacillus plantarum*-Mediated Intestinal Proteolytic Activity during Juvenile Growth

**Figure 5:**
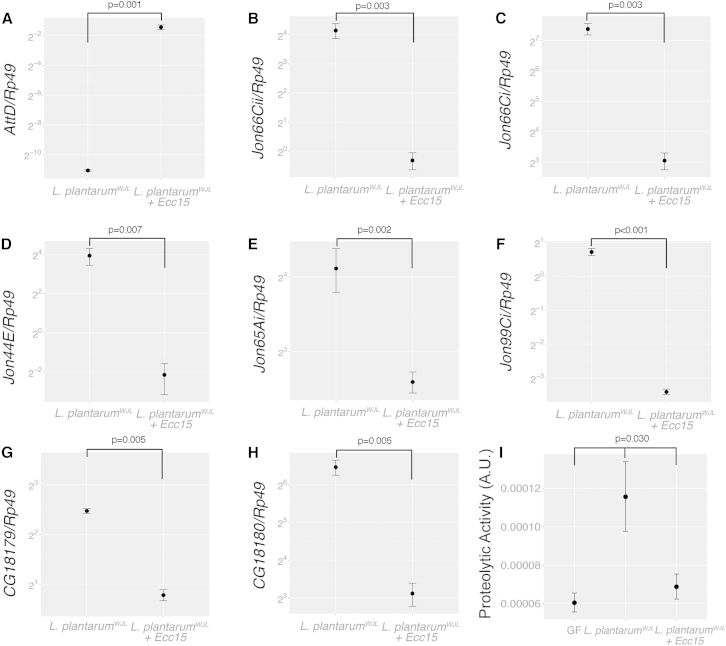
Foodborne Pathogen Infection Antagonizes *L. plantarum*-Mediated Intestinal Peptidase Expression and Activity during Juvenile Growth (A–H) Mean ± SEM of ΔCt^*gene*^*/*ΔCt^*rp49*^ ratios for (A) *Attacin-D*, (B) *Jon66Cii*, (C) *Jon66Ci*, (D) *Jon44E*, (E) *Jon65Ai*, (F) *Jon99Ci*, (G) *CG18179*, and (H) *CG18180* detected in midguts of wild-type larvae associated with *Lp*^*WJL*^ for 8 days AED. 8 hr prior midgut dissection, animals were infected with *Ecc15* (OD 100) or sham treated. Corrected p values obtained from comparison of group means by Student’s t test with Welch correction are indicated. (I) Proteolytic activity (mean ± SEM) in midguts dissected from size-matched GF (12 days AED) and *Lp*^*WJL*^-associated animals (8 days AED), sham-treated or 8 hr post-*Ecc15* infection. Proteolytic activity is expressed as a.u. p values obtained by one-way ANOVA are indicated. Results of Tukey’s post hoc pairwise comparisons confirm the statistical significance observed. See also Figure S2 and Table S1.

We recently reported the existence of a transcriptional switch upon foodborne infection in adults, where the induction of infection-mediated midgut genes occurs while several microbiota-mediated midgut genes are silenced (Erkosar et al., 2014). We therefore wondered if that phenomenon applies to *Lp*^*WJL*^-mediated gene regulation in the larval midguts. To test this hypothesis, we studied the expression of the peptidases in *Lp*^*WJL*^-associated larvae after an acute oral infection by the intestinal pathogen *Pectobacterium carotovorum spp. carotovorum* strain *15* (referred to as *Ecc15*) (Basset et al., 2003). As a control of the effective infection, we analyzed the expression of the antimicrobial peptide gene *Attacin-D* (*AttD*), which is a marker of the host intestinal immune response to this infection (Bosco-Drayon et al., 2012; Tzou et al., 2000). As expected, *AttD* is potently induced in the *Lp*^*WJL*^-associated larval midgut upon *Ecc15* infection (Figure 5A). In contrast, the expression of all seven *Lp*^*WJL*^-regulated peptidases was dramatically reduced upon infection (Figures 5B–5H). Accordingly, when we analyzed the intestinal proteolytic activity of *Lp*^*WJL*^-associated midguts after infection, we detected an almost complete inhibition of the *Lp*^*WJL*^ association effect, with proteolytic activity levels decreasing upon infection to levels similar to those seen on GF midguts (Figure 5I). Collectively, these results demonstrate that acute pathogen infection antagonizes the *Lp*^*WJL*^-mediated promotion of peptidase activity during juvenile growth.

### Pathogen Virulence Impedes *Lp*^*WJL*^-Mediated Juvenile Growth Promotion by Antagonizing *Lp*^*WJL*^*-*Mediated Enhancement of Host Protein Digestion Capacities

**Figure 6:**
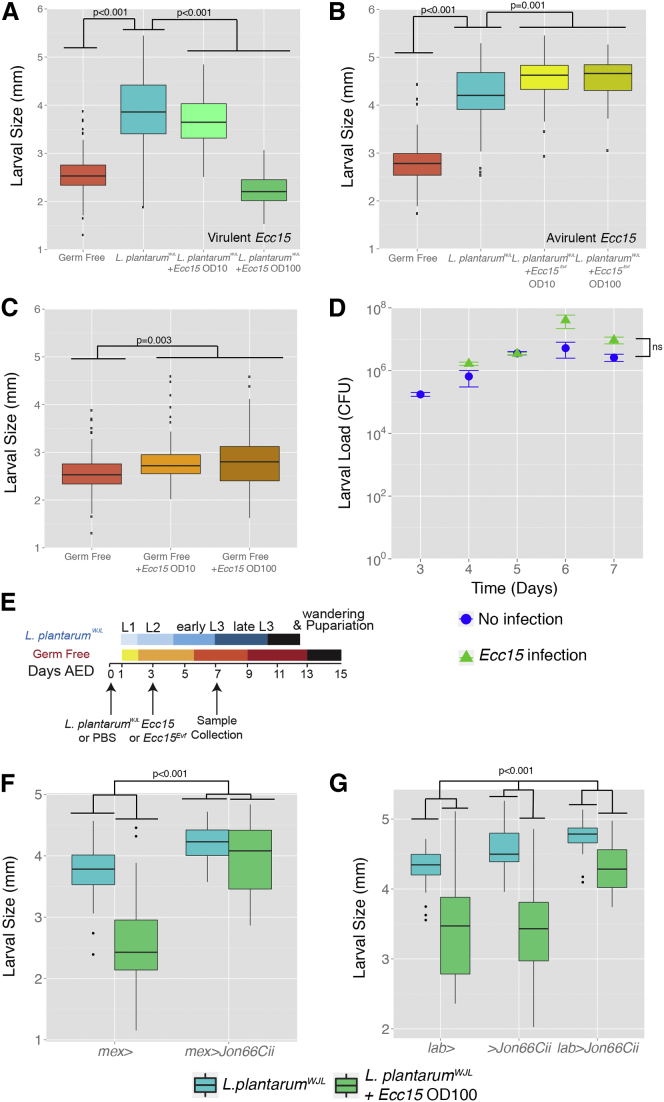
Pathogen Virulence Antagonizes *L. plantarum*-Mediated Juvenile Growth Promotion (A–D) Longitudinal size of larvae (boxplots, n > 54) 7 days AED on low-nutrition diet; larvae were kept GF or *Lp*^*WJL*^-associated at day 0 AED and at day 3 AED sham treated or infected with *Ecc15* ([A] and [C]) or with the avirulent mutant *Ecc15*^*Evf*^ (B). Timings of association, infections, and sampling are indicated in (D). AED, after egg deposition. (D) *Lp*^*WJL*^ loads in the larvae at the specified time points (days AED—*Ecc15* infection occurred at day 3 AED). (E) Experimental set-up for microbial association, infection and sampling. (F and G) Longitudinal size of larvae (Boxplots, n > 24) 7 days AED on low-nutrition diet; larvae were associated with *Lp*^*WJL*^ at day 0 AED and at day 3 AED sham treated or infected with *Ecc15*. Genotypes used: (F) *mex*>: *mex*-GAL4 - *mex*>*Jon66Cii*: *mex*-GAL4/+;;UAS-*Jon66Cii*-3xHA/+. (G) *lab*>: *lab*-GAL4 - *lab*>*Jon66Cii*: *lab*-GAL4/+;;UAS-*Jon66Cii*-3xHA/+ − > *Jon66Cii*: UAS-*Jon66Cii*-3xHA. p values obtained from Student’s t test with Welch correction, one-way ANOVA, and two-way ANOVA are indicated. Results of Tukey’s post hoc pairwise comparisons confirm the statistical significance observed in (F) and (G). See also Figures S3 and S4 and Table S1.

We next addressed the physiological consequence of an acute oral infection on *Lp*^*WJL*^-mediated juvenile growth promotion. To this end, we acutely infected a subset of GF and *Lp*^*WJL*^-associated larvae at day 3 AED with increasing quantities of virulent *Ecc15* or the avirulent mutant *Ecc15*^*Evf*^ (Basset et al., 2003) and measured the size of GF and *Lp*^*WJL*^-associated larvae on day 7 AED on the low-nutrition diet (Figures 6A, 6B, and 6E). Upon acute challenge with *Ecc15*, the enhanced longitudinal growth of *Lp*^*WJL*^-associated larvae is significantly altered in an *Ecc15* dose-dependent manner (Figure 6A). However, when *Lp*^*WJL*^-associated larvae were acutely infected with increasing quantities of the avirulent mutant *Ecc15*^*Evf*^, the *Lp*^*WJL*^-mediated benefit on larval growth persisted in all infection conditions tested (Figure 6B). Importantly, the alteration of *Lp*^*WJL*^-mediated benefit on larval growth by the acute *Ecc15* infection is not the result of a competition between *Lp*^*WJL*^ and *Ecc15*, since we detected similar amounts of *Lp*^*WJL*^ in the larvae pre- and post-infection (Figure 6D). Interestingly, GF larvae that were acutely infected with increasing quantities of *Ecc15* at day 3 AED (Figure 6C) or at day 5 AED (GF larvae on day 5 AED match day 3 AED *Lp*^*WJL*^-associated larvae in size; Figure S3) grew to their usual average size. Hence, our results demonstrate that the virulence of *Ecc15* specifically antagonizes the beneficial activity of *Lp*^*WJL*^ on juvenile growth but does not alter basal growth rate (i.e., as seen in GF). These results reveal that a selective physiological switch occurs in *Lp*^*WJL*^-associated juveniles when facing *Ecc15* foodborne infection whereby longitudinal growth is stunted. Next, we tested if *Ecc15* infection would alter *Lp*^*WJL*^-mediated benefit on larval growth in *Dredd* mutant larvae and observed that *Ecc15* infection also antagonizes *Lp*^*WJL*^-mediated benefit on the systemic growth in *Dredd* larvae. Therefore, the Imd/Relish signaling is not rate limiting for this physiological switch to occur (Figure S4).

Taken collectively, our results suggest that *Ecc15* infection impedes *Lp*^*WJL*^-mediated juvenile growth promotion by antagonizing *Lp*^*WJL*^*-*mediated enhancement of host protein digestion capacities. To formally validate this model, we forced the expression of *Jon66Cii* in the midgut of *Ecc15*-infected animals and tested if it would rescue *Lp*^*WJL*^-mediated juvenile growth promotion as expected under this hypothesis. To this end, we directed the expression of *Jon66Cii* either in all enterocytes of the larval midgut (Figure 6F) or just in the enterocytes of the acidic region of the middle midgut (Figure 6G) and assayed larval longitudinal growth of *Lp*^*WJL*^-associated animals at day 7 AED after an Ecc15 infection at day 3 AED (Figure 6E). In both cases, we observed a marked rescue of *Lp*^*WJL*^-mediated longitudinal growth when *Jon66Cii* was expressed under the control of the UAS/GAL4 system upon *Ecc15* infection (Figures 6F and 6G). These results therefore demonstrate that *Ecc15* infection alters *Lp*^*WJL*^-mediated growth by antagonizing intestinal protease expression.

## Discussion

**Figure 7:**
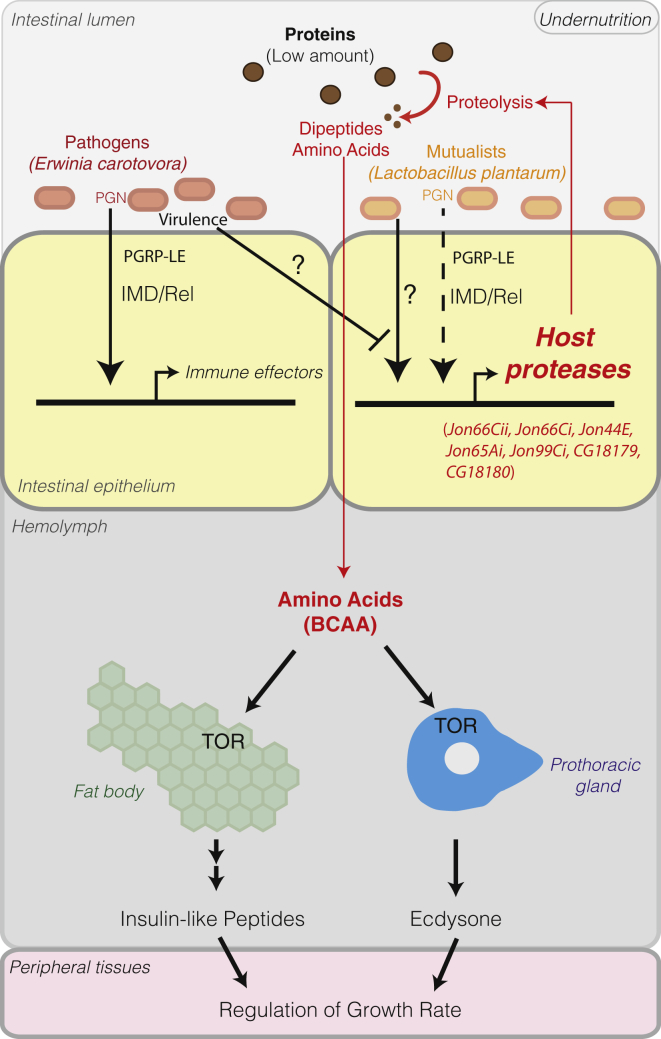
*Ecc15* Virulence Antagonizes *L. plantarum-*Mediated Enhancement of Host Protein Digestion Capacities Impeding *Drosophila* Juvenile Growth Promotion Upon undernutrition, the *Drosophila’s* mutualist *L. plantarum* promotes host proteases expression in enterocytes partly through the PGRP-LE/Imd/Relish signaling cascade. The resulting enhanced protease activity in the midgut increases the digestion and uptake of dietary proteins into dipeptides and AAs. AAs accumulation is sensed in endocrine tissues by the TOR kinase pathway and promotes increases production of dILPs and Ecdysone, which together drive systemic growth. Upon infection by the foodborne pathogen *Ecc15*, *L. plantarum*-mediated systemic growth promotion is attenuated. *Ecc15* virulence causes a transcriptional switch whereby immune genes expression is promoted in enterocytes while intestinal proteases expression is silenced.

Our results support the notion that the regulation of intestinal protease expression by commensal microbes plays an important role in the context of mutualist-mediated juvenile growth promotion upon undernutrition. We show that association of GF *Drosophila* juveniles with the mutualistic bacterial strain *Lp*^*WJL*^ promotes intestinal peptidase expression and activity, and such gut functionality is necessary and sufficient to sustain host systemic growth. In addition, we reveal that *Lp*^*WJL*^ association enhances dietary protein digestion—a feature characterized by increased levels of dipeptides and free AAs in *Lp*^*WJL*^-associated animals. This observation, together with our previous results positing that *Lp*^*WJL*^*-*mediated juvenile growth promotion genetically requires the AA transporter Slimfast and the kinase mTOR, indicates that *Lp*^*WJL*^ promotes juvenile growth via enhanced intestinal peptidase expression and increased AAs assimilation, which optimizes the activity of the mTOR kinase in endocrine tissues producing dILPs and Ecdysone, the two main drivers of systemic growth and maturation (Figure 7).

Recently, Yamada et al. (2015) showed that in the context of *Drosophila* aging, *Issatchenkia orientalis*, a *Drosophila* commensal fungus, extracts AAs directly from nutrient-poor diets and increases AAs flux to the host by being a direct food source for *Drosophila*. Hence, *I. orientalis* association increases the lifespan of undernourished flies. This work indicates that, upon undernutrition, commensal fungi can become a food source for their host and may impact host physiology by fortifying the diet (Yamada et al., 2015). Whether this concept applies to commensal bacteria in general and *Lp*^*WJL*^-mediated juvenile growth promotion in particular is an intriguing question. At this point, we cannot entirely rule out this possibility. However, we have previously demonstrated that other strains of *L. plantarum* (*Lp*^*IBDML1*^ and other strains), which can colonize efficiently the larval gut and the nutritional environment, do fail to mediate juvenile growth promotion (Storelli et al., 2011) (G.S. and F.L., unpublished data). Furthermore, in this study, we show that *Lp*^*WJL*^ association triggers a host transcriptional response in the *Drosophila* larval intestine to support essential gut functionalities necessary for systemic growth. These results support a model where *Lp*^*WJL*^ sustains *Drosophila* systemic growth via the promotion of specific host biological activities, rather than by being a mere food source for its host. In this light, our work now reveals that enhanced host intestinal peptidase expression should be considered as a host biological activity required for physiological benefit mediated by a mutualist association.

While we still lack a complete understanding of how *Lp*^*WJL*^ mediates the induction of intestinal peptidase gene expression, we found that some peptidase gene induction occurs in larval enterocytes and are partly regulated by the PGRP-LE/Imd/Relish cascade, a signaling pathway devoted to bacterial sensing by the host and previously associated with regulation of host immunity in the intestinal epithelium (Bosco-Drayon et al., 2012; Neyen et al., 2012). These results therefore demonstrate that in addition to regulating immune responses, the PGRP-LE/Imd/Relish cascade also influences dietary protein digestion in the midgut, albeit without being rate limiting to this biological process. Interestingly, we and others have previously shown that in adult midguts the Imd/Relish cascade is required for microbiota-induced expression of other digestive enzymes such as lipases, glycosyl-hydrolases, and alkaline phosphatase, thus raising the possibility that this signaling cascade may influence other digestive processes beyond dietary protein breakdown (Broderick et al., 2014; Erkosar et al., 2014). Taken together with the finding in this study, we propose that there are shared regulatory modules between the digestive and immune processes and that the PGRP-LE/Imd/Relish is at the cornerstone of this regulation. This observation echoes the theory proposing a common evolutionary origin for immunity and digestion in primitive guts based on the observation that many enzymes used as antimicrobial effectors during intestinal immune responses also play a role in digestion or share molecular ancestry with digestive enzymes (Broderick, 2015; Lemaitre and Miguel-Aliaga, 2013). Our study bolsters the theory by identifying some of the regulatory modules of these processes. Yet our results show that the regulation of intestinal protease expression is complex and that the PGRP-LE/Imd/Relish cascade only contributes partly to their regulation (Figure 7). Therefore, our work paves the way to further dissections of the regulatory networks underlying intestinal peptidase gene induction, which may shed light on regulatory cross talks between immunity and digestion.

The hypothesis of regulatory cross-talks between immunity and digestion is also supported by the observation that in adult midguts a transcriptional switch occurs upon foodborne bacterial infection, whereby several digestive enzymes expression is silenced while immune-related gene expression is induced (Buchon et al., 2009b). Interestingly, such transcriptional switch favors the induction of infection-mediated midgut genes at the expense of microbiota-mediated midgut genes (Erkosar et al., 2014). Here we discovered the same transcriptional switch in *Lp*^*WJL*^-associated juveniles upon infection with the pathogenic strain *Ecc15* by showing that *Lp*^*WJL*^-mediated intestinal protease induction is silenced upon *Ecc15* infection. Consequently, *Lp*^*WJL*^-mediated intestinal protease activity was suppressed. This striking observation allowed us to investigate the consequences of such transcriptional switch on the host physiology. *Lp*^*WJL*^ association to GF juveniles has a profound effect on host physiology by promoting juvenile longitudinal growth (Storelli et al., 2011). We now reveal that in *Lp*^*WJL*^-associated juveniles, *Ecc15* foodborne infection triggers a physiological switch stunting longitudinal growth. This switch is triggered by pathogen virulence, since infection with an *Ecc15* avirulent mutant (*Ecc15*^*Evf*^) did not trigger stunting of *Lp*^*WJL*^-associated juveniles. Surprisingly, age-matched or size-matched GF juveniles were not stunted by *Ecc15* foodborne infection, suggesting that *Ecc15* virulence selectively antagonized *Lp*^*WJL*^-mediated benefit to host longitudinal growth. These results therefore illustrate how the host adapts its physiology when facing either mutualists or pathogens by manipulating its digestive activity to influence its growth patterns during the juvenile phase of its life cycle.

Infection-associated host physiological switches were previously reported in *Drosophila*; *Listeria monocytogenes* or *Salmonella typhimurium* infections trigger anorexia (Ayres and Schneider, 2009), *Streptococcus pneumoniae* infection triggers the loss of circadian locomotor activity (Shirasu-Hiza et al., 2007), and *Mycobacterium marinum* and *Listeria monocytogenes* infections trigger metabolic switches and wasting (Chambers et al., 2012; Clark et al., 2013; Dionne et al., 2006), yet these studies were performed by injecting into the body cavity of adult flies a lethal dose of pathogens isolated from humans or other heterologous animals. These lethal infection models have furthered the understanding of the physiopathology of such lethal infection models but do not recapitulate the interaction between *Drosophila* and its natural microbial environment. In our experimental system, we used a natural pathogen and a natural mutualist of *Drosophila* so that we can reveal the physiological adaptability of *Drosophila* to its natural microbial environment, at least during the juvenile phase of its life cycle. With this setting, we find that pathogen virulence antagonizes a mutualist-mediated physiological benefit. Interestingly, it was previously established that, in the larval midgut, mutualists (including *Lp*^*WJL*^) promote intestinal immune tolerance while pathogens (including *Ecc15*) induce potent intestinal immune and tissue repair responses (Bosco-Drayon et al., 2012; Buchon et al., 2013). We therefore propose a model whereby, upon association with a mutualist microbe (such as *Lp*^*WJL*^), the host optimizes its juvenile growth and immune tolerance to more quickly reach the reproductive stage of its life cycle, while upon acute pathogen infection (such as *Ecc15* infection), the juvenile stunts its growth, allowing the triggered intestinal immune and tissue repair mechanisms to efficiently resolve the infectious episode that could be detrimental to its reproductive success at the adult stage. In this model, the PGRP-LE/Imd/Relish cascade activation and the regulation of intestinal peptidase expression stand as molecular cornerstones in these events (Figure 7).

Given the importance of the microbial environment in the ecological success and evolution of host species (McFall-Ngai et al., 2013) and the importance of immunity and juvenile growth in this context, our work, which illustrates the profound impact of the microbial environment on both traits, paves the way for future studies focusing on the adaptive value of the physiological switch triggered by pathogen virulence in the host.

## Experimental Procedures

### *Drosophila* Diets, Stocks, and Breeding

*Drosophila* stocks were cultured at 25°C with 12/12 hr dark/light cycles (light switch at 1:00 PM) on a yeast/cornmeal medium containing 50 g/l inactivated yeast as described in Erkosar et al. (2014). The low-nutrition diet was obtained by reducing the amount of inactivated yeast to 6 g/l. AEBSF (Sigma, ref. #A8456) and PIC (Sigma, ref. #P2714) were included in the diets at indicated concentration. GF stocks were established as described in Erkosar et al. (2014). *Drosophila y*,*w* flies were used as the reference strain in this work.

### Bacterial Strains

*Lactobacillus plantarum*^*WJL*^ (referred as *Lp*^*WJL*^) (Ryu et al., 2008), *Pectobacterium carotovorum spp. carotovorum*^*15*^ (referred as *Ecc15*), and *Pectobacterium carotovorum spp. carotovorum*^*15-Evf*^ (referred as *Ecc15*^*Evf*^) (Basset et al., 2003) were used in this study.

### Colonization and Infection of Larvae

40 embryos collected from GF females were transferred to a fresh low-nutrition medium poured in small petri dishes (ø = 5cm). *Lp*^*WJL*^ cells (7 × 10^7^ CFUs, 300 μl, OD_600_ = 0.5 in PBS) or sterile PBS were added directly on the embryos and the medium. For acute oral infection, petri dishes containing the low-nutrition diet + GF or *Lp*^*WJL*^ associated larvae were supplemented with 300 μl of *Ecc15* or *Ecc15*^*Evf*^ cells in PBS (OD_600_ = 10 or 100) at day 3 or 5 AED.

### Larval Size Measurements

*Drosophila* larvae were collected 7 days AED, washed in distilled water, transferred on a microscopy slide, killed with a short heat treatment (5 s at 90°C), mounted in 80% glycerol/PBS, and pictured under a Leica stereomicroscope M205FA. Larval longitudinal size (length) was quantified using ImageJ software (Schneider et al., 2012).

### Bacterial Loads Analyses

Bacterial loads were quantified by plating serial dilutions of lysates obtained from five individuals on nutrient agar (MRS). Biological triplicates were collected for each experimental condition. Homogenization of the samples was performed using the Precellys 24 tissue homogenizer (Bertin Technologies) and 0.75–1 mm glass beads in 500 μl of PBS.

### RNA Extraction and qPCR Analysis

RNA extraction of three biological replicates of ten midguts (foregut, hindgut, and malphigian tubules removed) for each condition was performed as described in Erkosar et al. (2014). qPCR was performed using gene-specific primer sets (sequences provided in Table S2) and as described in Erkosar et al. (2014). Results were represented either as the value of ΔCt^*gene*^*/*ΔCt^*rp49*^ ratios or as the relative fold induction of the ΔCt^*gene*^*/*ΔCt^*rp49*^ ratios among conditions tested.

### Azocasein Assay

Three biological replicates of ten midguts per condition were dissected in 50 μl of PBS and homogenized as for RNA extraction. 10 μl of sample were mixed with 300 μl of Azocasein solution (2.5 mg/ml in water, Sigma, ref. #A2765) and processed according to supplier’s instructions. For an extended protocol, see Supplemental Experimental Procedures.

### Metabolomics

Axenic embryos were inoculated with PBS or 10^8^ CFUs of *Lp*^*WJL*^ and reared on low-nutrition diet. Larvae were sampled at day 2.5 AED, snap frozen, and sent to Metabolon Inc. (http://www.metabolon.com). Five biological replicates were used containing each approximately 300 early L2 larvae of the same size. Samples were then extracted, normalized, and prepared for analysis using Metabolon’s standard solvent extraction method. The extracted samples were split into equal parts for analysis with GC/MS and LC/MS/MS. Compounds were identified by comparison to library entries of purified standards or recurrent unknown entities.

### Statistical Analysis

Statistical calculations were made using R; details are provided in Supplemental Experimental Procedures. Detailed statistics are provided for each panel figures in Table S1.

## Author Contributions

F.L. supervised the work. B.E., G.S., and F.L. designed the experiments. B.E., G.S., M.M., L.B., and N.B. performed experiments. B.E. performed statistics. F.L., B.E., and G.S. analyzed data. B.E. and F.L. wrote the paper.

## Supplemental Information

Document S1. Figures S1–S4, Table S1, and Supplemental Experimental Procedures

Table S2. Primers Sequence for RT-qPCR. Related to Figures 1 and 4

Document S2. Article plus Supplemental Information
