# Supplementary material for: Pathogen Virulence Impedes Mutualist-Mediated Enhancement of Host Juvenile Growth via Inhibition of Protein Digestion
Source: Cell Host Microbe. 2015 Oct 14;18(4):445–55. doi: 10.1016/j.chom.2015.09.001 (PMC4617634; doi:10.1016/j.chom.2015.09.001)
Supplement: Document S1. Figures S1–S4, Table S1, and Supplemental Experimental Procedures [file mmc1.pdf]

Cell Host & Microbe, Volume *18*

Supplemental Information

Pathogen Virulence Impedes Mutualist-Mediated Enhancement of Host Juvenile Growth via Inhibition of Protein Digestion

Berra Erkosar, Gilles Storelli, Mélanie Mitchell, Loan Bozonnet, Noémie Bozonnet, and François Leulier

Supplemental Information

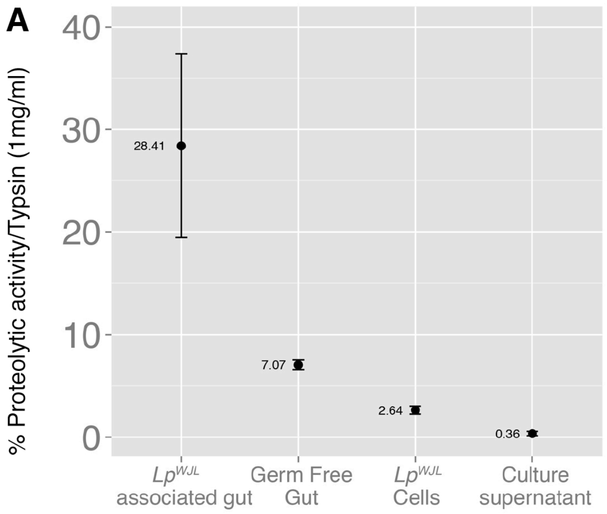

Supplementary Figure 1, related to Figure 2: The difference in the proteolytic activity represented in Fig 2A cannot be explained by the mere proteolytic activity of *L. plantarum*^{WJL} on its own. Dotplots representing the relative proteolytic activity obtained from larval intestines either germ free or containing a maximum of 10^7 *L. plantarum*^{WJL} cells, *L. plantarum*^{WJL} culture pellet (10^9 cells), and an over-night *L. plantarum*^{WJL} culture supernatant (400ul). All values were relativized (%) to the proteolytic activity obtained from 1mg/ml Trypsin solution. Values from gut samples were not normalized to total protein quantity for the consistency within the analysis and represent absolute values obtained from 10 dissected guts.

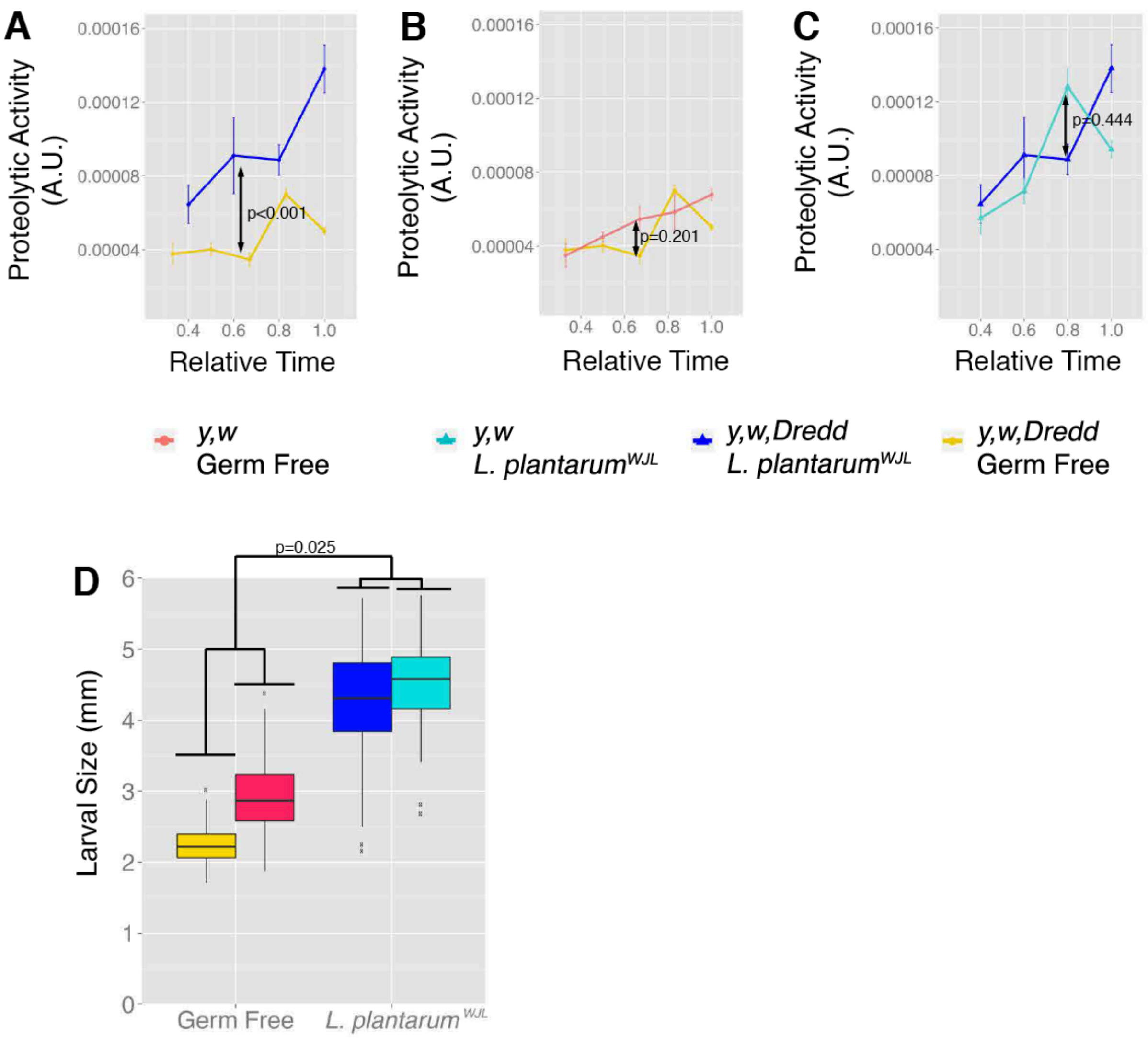

Supplementary Figure 2, related to Figure 5: Increased proteolytic activity and growth promotion is observed upon *L. plantarum*^{WJL} association in *Dredd* mutants similarly to wild type larvae.

(**A-C**) Line graphs representing mean±SEM units of proteolytic activity measured by azocasein assay normalized to total protein quantity for each sample. Each condition at every time point contains at least three biological replicates of ten larval guts dissected. (**D**) Boxplots (n>61) represent the larval size in germ free and *L. plantarum*^{WJL} associated larvae, 7 days after egg deposition in wild type (*y,w*) and *Dredd* mutant (*y,w,Dredd)* genotypes. p values obtained by two-way ANOVA are indicated.

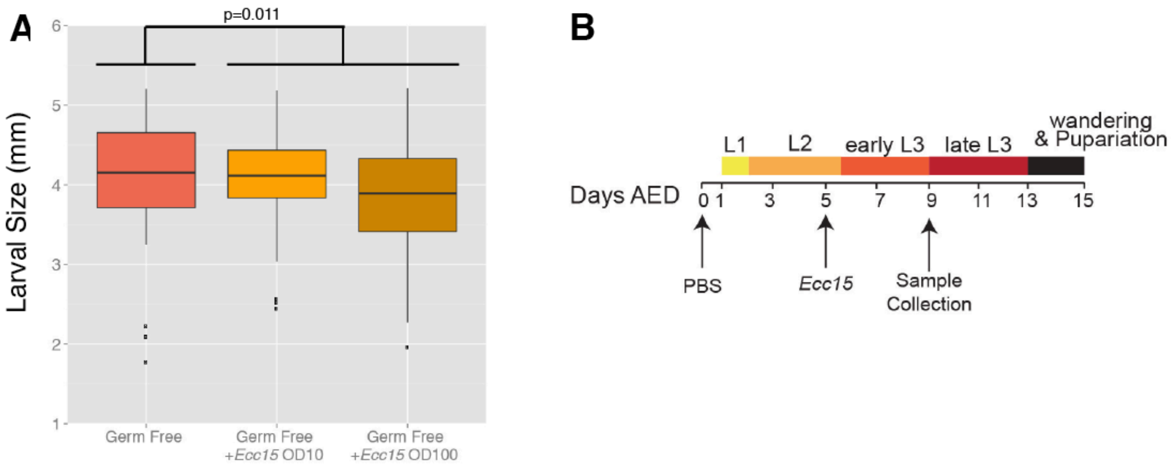

Supplementary Figure 3, related to Figure 6: *Ecc15* infection does not alter growth of GF animals

(A) Boxplots (n>55) representing the size of GF larvae at 9 days AED that were sham treated or infected with *Ecc15* at 5 days AED (at this time GF larvae are size matched with 3 days old Lp^{WJL}-associated larvae). (**B**) Timings of association, infections and sampling are indicated. p-values obtained by one-way ANOVA are indicated. A statistically significant size reduction is observed, however the growth inhibition observed in this setting is biologically not relevant as compared to the effect observed on Lp^{WJL}-associated larvae.

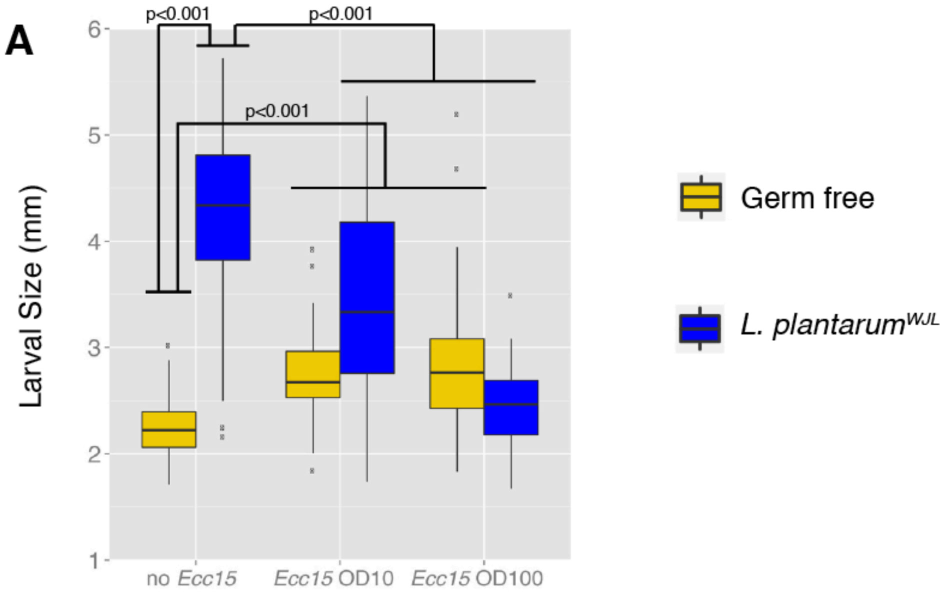

Supplementary Figure 4, related to Figure 6: *Ecc15* suppresses *L.plantarum*^{WJL} mediated growth promotion in *Dredd* mutants as in wild-type larvae

(**A**) Boxplots (n>41) represent the size of 7 days old, germ free or *L. plantarum*^{WJL} associated, *Dredd* (*y,w,Dredd)* larvae which were infected with increasing quantities of *Ecc15* (DO 10/100) 3 days after egg deposition. p-values obtained by Student's T-Test with Welch correction and one-way ANOVA are indicated.

Supplementary Table 1, related to all Figures.

Statistical tests and p values for data represented in all Figures.

Supplementary Table 2, related to Figure 1 and 4.

Primers sequence for RT-qPCR

Gene Name	Forward Primer	Reverse Primer
Jon66Cii	aaactgaccccggtccac	cctcctcagccggatagc
Jon66Ci	cgtcggtctgggtttcag	acccactcgttggagatgat
Jon44E	acagcgctaaccatgtgct	ggtgtactgggcctcgtg
Jon65Ai	caacaactaccaaggctggtg	gccctcatcggaggtctt
Jon99Cii	tccataatcggacacacttgg	cagtgaagcctcatcagcac
CG18179	accgatggcaaatcctctt	gcgttgtcatgggtaacga
CG18180	ctacgtggagatccactacgg	acggacggtctgcctgta
PGRP-SC1	aagcgatcgtcaactatt	gagagccactttggaaacca
AttD	gtcactagggttcctcag	gccgaaatcggacttg

Supplementary Methods

Drosophila diets, stocks and breeding

Fresh food was prepared every week to avoid desiccation, and no yeast paste was added to the medium. GF stocks were established by bleaching and cultivating embryos on autoclaved conventional medium supplemented with a cocktail of four antibiotics (final concentration: 50 µg/mL ampicillin, 50 µg/mL kanamycin, 15 µg/mL erythromycin, 50 µg/mL tetracyclin) for a few generations. In experimental settings GF animals were used without antibiotics. Germ-free-ness was routinely tested by plating serial dilution of animal lysates on nutrient agar plates. The following *Drosophila* lines were used: *y,w* - *y,w,Dredd*^{F64} (Leulier et al., 2000) ; *y,w, PGRP-LE*^{112} (Takehana et al., 2004), *labial*-GAL4 (Hoppler and Bienz, 1994) (BL#43651) , *mex*-GAL4 (Phillips and Thomas, 2006), UAS-*Jon66Cii*-3xHA (Bischof et al., 2013) and UAS-*Pirk* (Aggarwal et al., 2008).

Bacterial culture conditions

Lp^WJL was cultivated in Man, Rogosa and Sharpe (MRS) broth medium (Difco, ref. #288110) over night at 37°C without shaking and *Ecc15* in Luria-Bertani broth medium (Difco, ref. #244610) over night at 30°C with agitation. Note that *Ecc15* does not grow in liquid MRS or on MRS-agar plates.

Azocasein Assay

10µl of sample were mixed with 300µl of Azocasein solution (2,5mg/ml in water, Sigma, ref.#A2765). Samples were incubated for 4 hours at 37°C. 300µl of 20% Trichloroacetic acid (TCA, Sigma, ref.#T6399) was used to stop the enzymatic reaction and precipitate the undigested Azocasein. Recombinant trypsin (serial dilutions from 1mg/ml stock solution in PBS, Sigma, ref.#T1426) was used to calibrate the assay and as a positive control in each reaction. The reaction blank was generated using 10µl of 1mg/ml of Trypsin mixed with TCA prior to incubation. Samples were centrifuged for 5 minutes at 13 krpm. 600µl of the supernatant was transferred to cuvettes and completed with 200µl 2M NaOH to reveal the color. Absorbance at 440nm was measured using a spectrophotometer. The reference arbitrary unit of proteolytic activity.ml^{-1} = 0.01 A^{440} units / hour. Protein concentration of samples was measured using a Nanodrop apparatus, reading the absorbance at 280nm and was used for normalization.

Statistical analysis

For pairwise comparisons Student's T-Test was applied with Welch correction. For group comparisons, one or two-Way Analysis of Variance (ANOVA) was applied regarding the experimental design. Application conditions for ANOVA were tested using Shapiro-Wilk normality test and Levene test in order to check the equal variances

among datasets. Log transformation was applied when it was necessary. Tukey's HSD test was used as a post-hoc test for pairwise comparisons post-ANOVA (multcomp package). Upon more than three comparisons p-values were corrected using Holm-Bonferroni method. For multivariate analysis, Multivariate ANOVA (MANOVA, when applicable) and Principal Component Analysis (PCA) were performed (ade4 package). Student's T test with Welch correction and/or Wilcoxon's rank sum tests were performed to determine the significance of differences in metabolites levels between Lp^{WJL}-associated and GF samples. The False Detection Rate (FDR) for a given compound is estimated using the *q*-value (Storey and Tibshirani, 2003).

Supplementary References

Bischof, J., Bjorklund, M., Furger, E., Schertel, C., Taipale, J., and Basler, K. (2013). A versatile platform for creating a comprehensive UAS-ORFeome library in Drosophila. Development *140*, 2434-2442.

Schneider, C.A., Rasband, W.S., and Eliceiri, K.W. (2012). NIH Image to ImageJ: 25 years of image analysis. Nature methods *9*, 671-675.

Storey, J.D., and Tibshirani, R. (2003). Statistical significance for genomewide studies. Proceedings of the National Academy of Sciences of the United States of America *100*, 9440-9445.
